# Shortened Measurement Time of Functional Visual Acuity for Screening Visual Function

**DOI:** 10.1155/2019/8950418

**Published:** 2019-09-10

**Authors:** Yuki Hidaka, Sachiko Masui, Yasuyo Nishi, Masahiko Ayaki, Minako Kaido, Masaru Mimura, Kazuo Tsubota, Kazuno Negishi

**Affiliations:** ^1^Department of Ophthalmology, Keio University School of Medicine, 35 Shinanomachi Shinjuku-ku, Tokyo, Japan; ^2^Department of Neuropsychiatry, Keio University School of Medicine, 35 Shinanomachi Shinjuku-ku, Tokyo, Japan

## Abstract

The functional visual acuity test which is the average of the visual acuities measured during a specific time frame (standard, 60 seconds) has been used recently to assess the visual function in various conditions. The availability of a shorter version of the functional visual acuity test promises to be patient friendly in that it is a simple screening test performed in a shorter period of time than the standard test. The results of measurements of the FVA test between the 30-second measurement time (short-version FVA test) and the standard 60-second measurement are compared, and the feasibility of the short-version FVA test instead of the standard FVA test is investigated. Twenty-eight healthy volunteers (25 men and 3 women) were enrolled in this prospective observational study. All subjects underwent measurement of the binocular distance-corrected visual acuity and the binocular distance-corrected FVA with the 60-second and 30-second measurement times. The interchangeability of the corrected-distance FVA, maximal VA, visual maintenance ratio, and average response time in the short-version and the standard FVA tests was evaluated using the Bland–Altman method, and the results showed agreements of the two tests except for the minimal VA. The short-version FVA test is equivalent to the standard method except for evaluating the visual acuity fluctuations and promises to be a simple visual screening test that can be performed in a shorter time.

## 1. Introduction

The conventional visual acuity test traditionally has been accepted widely to assess and screen visual function. Many professions including professional driving, flying, and quality control require a specific visual acuity level. [1] However, the conventional visual acuity test is not always adequate to evaluate the quality of vision and results in insufficiently reflected visual function in daily life.

The functional visual acuity test, which first was developed to detect impaired visual function in dry eye syndrome, [2] is the average of the visual acuities measured during a specific time frame (standard, 60 seconds) and has been used recently to assess the visual function in various conditions [3–18]. These studies showed that the functional visual acuity is more sensitive for detecting changes in visual function than the conventional visual acuity and can evaluate the visual function in daily life more precisely. Moreover, functional visual acuity reflects visual function related to quick recognition of the target, according to the measurement condition requiring a judgment time within the limited set time [19], and has been reported to be one of the promising candidates of the new standard of the vision screening test for driver's licenses [11, 20].

However, in light of the efficacy of the screening test in large populations such as vision testing for driver's licenses, the standard functional visual acuity test with a measurement time of 60 seconds still may be time-consuming, and the measurement time should be shortened as much as possible.

In the current study, we compared the results of the functional visual acuity test obtained using a 30-second measurement time (short-version functional visual acuity) with the standard 60-second functional visual acuity test and investigated the feasibility of using the short-version functional visual acuity test instead of the standard functional visual acuity test.

## 2. Methods

### 2.1. Study Design

This was a prospective study performed at the Department of Ophthalmology, Keio University School of Medicine. The institutional review board of Keio University School of Medicine approved the study (approval number, 20150077). All subjects provided written informed consent and consented to the publication of the case details. The study adhered to the tenets of the Declaration of Helsinki.

### 2.2. Subjects

Twenty-eight healthy volunteers (25 men and 3 women) were enrolled from the general population of our department. The inclusion criterion was a distance-corrected visual acuity above 20/20. The exclusion criterion was the presence of systemic or ocular diseases except for refractive errors that affect visual function. Participants were screened and enrolled based on visual acuity examination and self-reported systemic and ocular diseases.

### 2.3. Visual Acuity and Functional Visual Acuity Measurements

**Figure 1:**
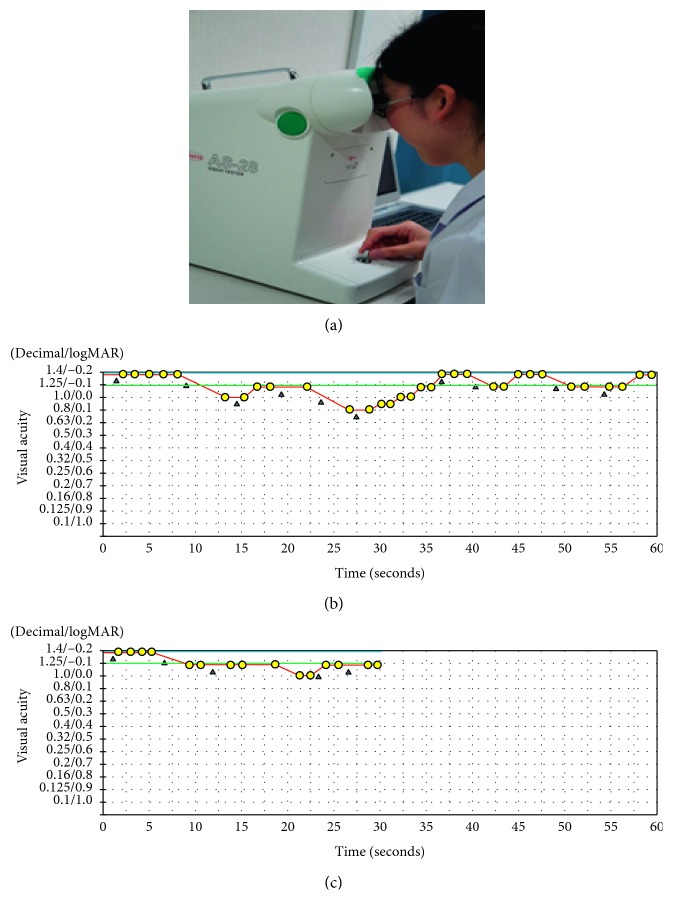
The functional visual acuity **(**FVA) measurement system and results of typical cases. (a) The FVA measurement system. (b) A representative printout of the results of the FVA test using the standard method. The blue line denotes the Landolt corrected visual acuity. The red line shows the timewise changes in the visual acuity during testing. The green line denotes the mean logarithm of the minimum angle of resolution (logMAR) visual acuity during the measurement (defined as the FVA). The yellow circles indicate the number of correct responses; the blue triangles indicate spontaneous blinks. FVA (logMAR), −0.09; visual maintenance ratio, 0.95; and maximal/minimal logMAR visual acuities, −0.18/0.10, respectively; average response time, 1.39 seconds. (c) A representative printout of the results of the FVA test using the short-version method in the same subject as in B FVA (logMAR), −0.10; visual maintenance ratio, 0.95; maximal/minimal logMAR visual acuities, −0.18/0.00, respectively; average response time, 1.30 seconds.

All subjects underwent measurement of the binocular distance-corrected visual acuity using Landolt vision charts and the binocular distance-corrected functional visual acuity using the AS-28 functional visual acuity measurement system (Kowa, Aichi, Japan) (Figure 1(a)).

The functional visual acuity measurement system was used to examine the timewise changes in continuous visual acuity during a given measurement time. The Landolt optotypes are presented in the device for a maximal period of 2 seconds, and their changes in size depend on the correctness of the subject's responses. The measurement starts with the best-corrected Landolt visual acuity A, which is the baseline visual acuity for each participant. The Landolt optotypes decrease in size automatically with correct answers; when the responses are incorrect or subjects do not response within 2 seconds, larger optotypes are presented automatically. Subjects delineate the orientation of the automatically presented Landolt rings from the baseline visual acuity using a joystick. [15] The system can measure visual acuity levels from 30/20 to 20/200. The test was performed with spontaneous blinking. In the current study, the functional visual acuities during 60 and 30 seconds were recorded for one subject (standard- and short-version functional visual acuities, respectively). The order of the measurements with the two tests was selected randomly. The subjects practiced for 60 seconds before the measurement to eliminate the effect of unfamiliarity with the test.

The continuous visual acuity changes were plotted in yellow (Figures 1(b) and 1(c)). The functional visual acuity measurement included several evaluation indices: the functional visual acuity, maximal and minimal visual acuities, visual maintenance ratio, and average response time. The functional visual acuity was defined as the average of all visual acuity values measured over time. The visual maintenance ratio was calculated as follows: visual maintenance ratio = (lowest logarithm of the minimum angle of resolution visual acuity score–functional visual acuity)/(lowest logarithm of the minimum angle of resolution visual acuity score–baseline visual acuity). The functional visual acuity was measured with the best correction.

### 2.4. Tear Function Evaluation

The tear function was evaluated in all subjects because it affects the functional visual acuity [2, 21, 22]. The standard Schirmer's test without topical anesthesia, the standard tear breakup time measurement, and the fluorescein corneal staining test with a score ranging from 0 to 9 points [23] were performed for all subjects. Standardized strips of the filter paper (Showa Yakuhin, Tokyo, Japan) were placed in the lateral canthus away from the cornea and left in place for 5 minutes with the eyes closed. The breakup time was measured after instillation of fluorescein sodium in the conjunctival sac using test filter paper. The interval between the last complete blink and the appearance of the first corneal black spot in the stained tear film was measured three times, and the mean value of the measurements was calculated.

### 2.5. Statistical Analysis

Comparisons of the corrected-distance functional visual acuity, maximal visual acuity, minimal visual acuity, visual maintenance ratio, and average response time in the 30-second and 60-second groups were performed using the Wilcoxon signed-rank test. Spearman's rank correlation analysis was used to study correlations among the distance-corrected functional visual acuity, maximal visual acuity, minimal visual acuity, visual maintenance ratio, and average response time in each group. *P* < 0.05 was considered significant. The Bland–Altman method was used to evaluate the interchangeability of the corrected-distance FVA, maximal VA, visual maintenance ratio, and average response time in the 30-second and 60-second groups. All statistical analyses were performed using IBM SPSS Statistics 22.0 software (IBM, Armonk, NY).

## 3. Results

The mean subject age was 34.6 ± 7.9 years (standard deviation) (range, 22–51 years). The mean distance-corrected visual acuity was −20 ± 0.07 (range, −0.30‐−0.08), and the mean manifest refraction spherical equivalent was −3.8 ± 3.3 diopters (range, 0.0∼−11.5).

### 3.1. Tear Function Evaluation

The mean Schirmer's test and breakup time values were 10.2 ± 10.3 mm (range, 1∼35) and 5.1 ± 2.6 seconds (range, 1∼12), respectively. The corneal staining test was 0.2 ± 0.4 (range, 0∼1).

Eight (14.3%) eyes of four subjects had normal tear functions, i.e., a BUT exceeding 5 seconds, a Schirmer test score exceeding 5 mm, and a keratoconjunctival vital staining score below 3 points; however, no subjects had dry eye symptoms. Thirty-five (60.3%) of 56 eyes had a BUT of 5 seconds. Only 24 (42.9%) of 56 eyes had a Schirmer test score of 5 mm or less. However, no patients in either group met the new Japanese dry eye diagnostic criteria [24].

### 3.2. Functional Visual Acuity Measured Using the Standard- and the Short-Version Tests

Table 1 shows the parametric values of the standard- and short-version functional visual acuity tests. There were no significant differences in the parameters between the standard- and short-version functional visual acuity tests except for the minimal visual acuity.

**Figure 2:**
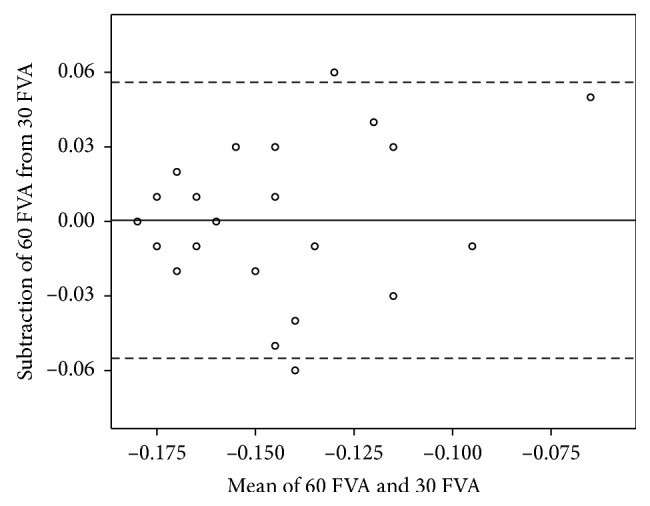
The Bland–Altman plot shows the agreement between the FVA in the logarithm of the minimum angle of resolution (logMAR) measured using the standard- and short-version methods. The solid line shows the mean difference, and the dashed lines show the 95% limits of agreement. Several plots overlap. 30 FVA = FVA measured using the short-version method; 60 FVA = FVA using the standard-version method.

**Figure 3:**
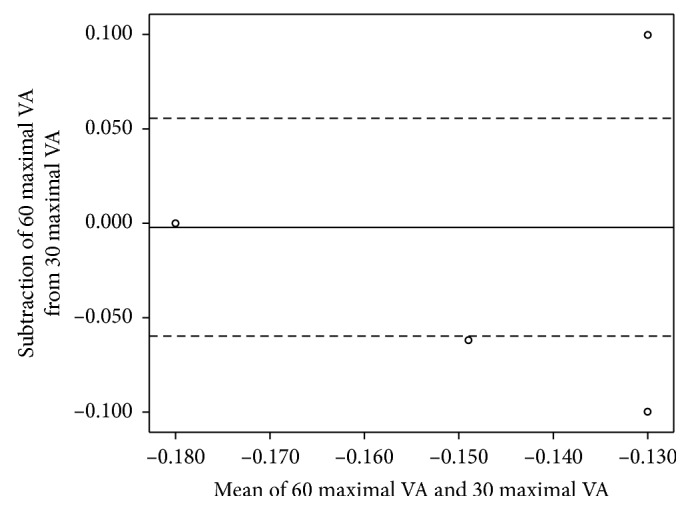
The Bland–Altman plot shows the agreement between the maximal visual acuity in logarithm of the minimum angle of resolution (logMAR) measured using the standard- and short-version methods. The solid line shows the mean difference, and the dashed lines show the 95% limits of agreement. Several plots overlap. 30 maximal VA = maximal VA measured using the short-version method; 60 maximal VA = maximal VA using the standard-version method.

**Figure 4:**
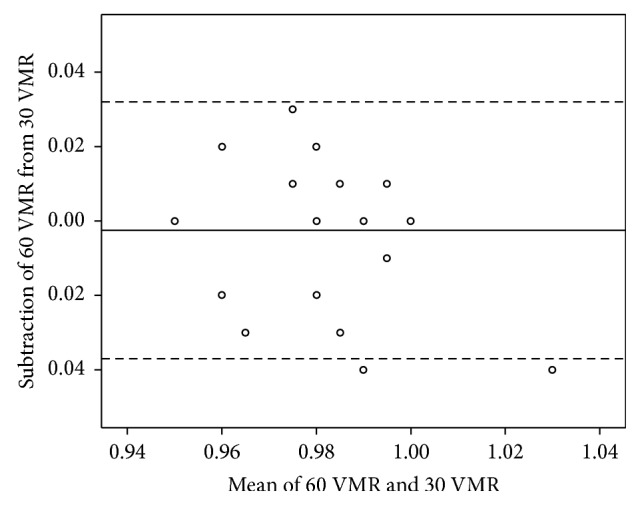
The Bland–Altman plot shows the agreement between the visual maintenance ratio (VMR) measured using the standard- and short-version methods. The solid line shows the mean difference, and the VMR dashed lines show the 95% limits of agreement. Several plots overlap. 30 VMR = VMR measured using the short-version method; 60 VMR = VMR measured using the standard-version method.

**Figure 5:**
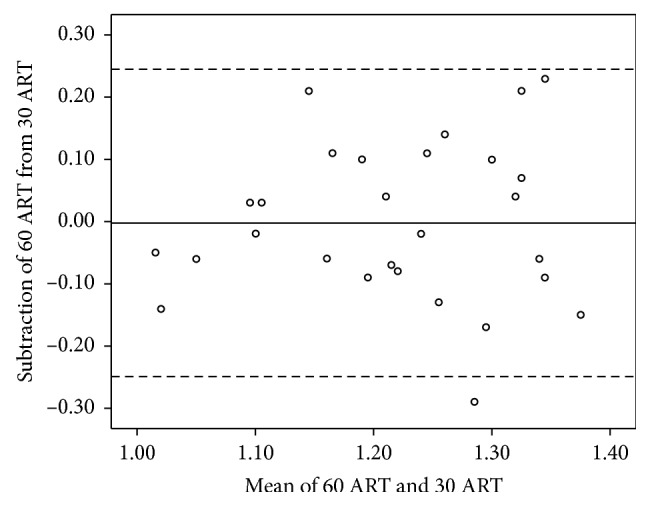
The Bland–Altman plot shows agreement between the average response time (ART) measured using the standard- and short-version methods. The solid line shows the mean difference, and dashed lines show the 95% limits of agreement. Several plots overlap. 30 ART = ART measured using the short-version method; 60 ART = ART measured using the standard-version method.

The agreements of the FVA, maximal VA, visual maintenance ratio, and average response time between the standard test and the short-version test are shown in Figures 2–5.

### 3.3. Factors Affecting the Minimal Visual Acuity

**Figure 6:**
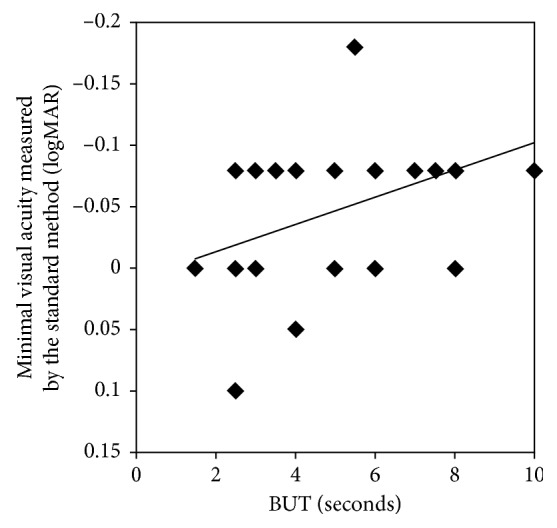
The relationship between the BUT and the minimal VA of the standard FVA test. The BUT and the minimal VA of the standard FVA test are correlated significantly (*r* = −0.416; *P*=0.028). logMAR = logarithm of the minimum angle of resolution.

To explore the cause of the difference in the minimal visual acuity between the standard- and short-version functional visual acuity tests, we conducted stepwise multiple regression analysis to investigate the predictors of the minimal visual acuity on the independent variables (age, sex, distance-corrected visual acuity, results of the Schirmer's test, breakup time measurement, and the fluorescein corneal staining test). The analysis showed that the breakup time (*β* = −0.482; *P*=0.007) and the distance-corrected visual acuity (*β* = 0.354; *P*=0.042) were the significant factors that affected the minimal visual acuity of the standard functional visual acuity test. The relationship between minimal visual acuity of the standard method and the breakup time is shown in Figure 6 (*r* = −0.416; *P*=0.028). In contrast, there was no significant correlation between the breakup time and the minimal visual acuity of the short-version method (*r* = −0.058; *P*=0.768).

## 4. Discussion

In the current study, we compared the results of the short-version functional visual acuity test with those of the standard test. The results of the two tests showed agreement except for the minimal VA, which suggested that the short-version FVA test might be a more useful screening tool for measuring visual function than the standard FVA test because of the shorter measurement time.

Previous studies have reported that the functional visual acuity test can detect detailed visual function compared with the conventional visual acuity test. The functional visual acuity test first was applied to assess visual impairment in dry eye syndrome, and it has been used to evaluate detailed visual function in various conditions including eye drop instillation [25], eye ointment instillation [26], laser in situ keratomileusis [27], retinal disease [8, 10], posterior capsular opacification [7, 28], early cataract [3, 4], astigmatism [5], soft contact lens wear [6], early presbyopia [13], and glaucoma [9, 29]. These reports have shown the usefulness of the functional visual acuity test for detecting decreased visual function that cannot be detected by the conventional visual acuity test and for identifying visual impairment in eyes with good visual acuity.

The FVA has been measured previously using the 10- to 30-second measurement times [16, 30] although the measurement conditions differed somewhat from the current conditions. Ishida et al. reported that the FVA during the 10-, 20-, and 30-second blink-free periods using topical anesthesia to minimize discomfort and prevent reflex tearing and blinking during measurement degraded as the measurement time increased, even in normal subjects [30]. Ishioka et al. investigated the effect of eye drop instillation on visual function using an old version of the FVA test for 30 seconds and reported that the transient, short-term blurring of vision caused by viscous eyedrops was detectable using the FVA test [16]. In the study of Ishioka et al., the patients were permitted to blink freely during the measurements and topical anesthesia was not administered; that is, the measurement conditions were similar to those in the current study. However, a detailed evaluation of the parameters including the maximal and minimal VAs, visual maintenance ratio, and average response time was not performed, and the measurement time was not mentioned.

The spontaneous blink rate in normal subjects has been reported to be 15.54 ± 13.74 (mean ± standard deviation) times/min that decreases during visual display terminal use to 5.34 ± 4.53 times/min [31]. In other words, even in normal subjects, the blink rate varies individually and some may blink only once or twice/minute minimally. Considering this, we chose 30 seconds for the measurement time because the FVA test might not detect visual function fluctuations resulting from changes in the ocular surface condition if the measurement time was less than 30 seconds.

The results showed that no cases in either group met the new Japanese dry eye diagnostic criteria [24] However, this does not mean that subjects with a short BUT should be excluded. The new Japanese dry eye diagnostic criteria are as follows: (1) the presence of dry eye symptomatology, (2) presence of either qualitative or quantitative disruption of the tear film (Schirmer test ≤5 mm or BUT ≤5 seconds), and (3) presence of conjunctivocorneal epithelial damage (a fluorescein staining score of 3 points, a Rose Bengal staining score of 3 points, or a lissamine green staining score of 3 points). The presence of all three criteria is required to establish a diagnosis of dry eye [24]. In the current study, only four (14.3%) eyes of 16 subjects had normal tear functions, i.e., the BUT exceeding 5 seconds, a Schirmer test exceeding 5 mm, and keratoconjunctival vital staining score below 3 points, although no subjects had dry eye symptoms. In the current study, 35 (60.3%) of 56 eyes had a BUT of 5 seconds or less; only 24 (42.9%) of 56 eyes had a Schirmer test score of 5 mm or less. Kaido et al. previously assessed the tear function of their subjects [32] whose mean age was similar to that of the current study. According to that report, of the 146 eyes of 73 subjects (50 men and 23 women; mean age 30.6 years) without dry eye symptoms, only 46 (31.5%) eyes met all of the criteria for normal tear function. Regarding the BUT, nearly half of the subjects without dry eye symptoms had a short BUT. The percentage of patients with marginal dry eyes in the current study was higher than the previous reports, and this should be considered when evaluating the results.

In the current study, the minimal visual acuity, which reflects visual fluctuations, differed significantly between the standard- and short-version functional visual acuities and indicated that a 30-second test is insufficient to evaluate the visual acuity fluctuations in daily life. However, the clinical relevance of the minimal VAs remains unclear because previous studies have evaluated only the functional visual acuity and the visual maintenance ratio, except for a study that reported that the numbers of subjects with functional visual acuity and minimal visual acuity scores less than 0.7 in decimal visual acuity were significantly higher in older drivers than in younger drivers, and the minimal visual acuity scores were correlated significantly with the subjective visual performance while driving during the day. [20] In the current results, the minimal visual acuity was correlated significantly with the breakup time only when using the standard method but not when using the short-version method. This suggested that 30 seconds might not be sufficiently long to evaluate visual fluctuations due to ocular surface instability. Further investigations should be performed to determine the clinical importance of the minimal VAs.

A limitation of the current study was that it included the subjects with marginal dry eyes. The relationship between the results of the standard- and short-version methods should be evaluated further in a study that includes subjects with a wider age range and ocular conditions including an ocular surface condition by calculating the sensitivity and specificity. In addition, most subjects in the current study were men. Another study should be performed to evaluate the differences between men and women.

## 5. Conclusions

The short-version functional visual acuity test is as useful as the standard test, except for evaluating visual acuity fluctuations, and promising as a simple vision screening test that can be performed in less time.

## Figures and Tables

**Table 1:** Comparison of the parameters of the functional visual acuity (FVA) test between the standard test and the short-version test (*n* = 28).

Parameters	Standard method (mean ± standard deviation)	Short-version method (mean ± standard deviation)	*P* value^*∗*^
Distance-corrected FVA (logMAR)	−0.147 ± 0.030	−0.146 ± 0.035	0.931
Maximal visual acuity (logMAR)	−0.174 ± 0.022	−0.176 ± 0.019	0.785
Minimal visual acuity (logMAR)	−0.048 ± 0.062	−0.079 ± 0.069	0.036
Visual maintenance ratio	0.99 ± 0.02	0.98 ± 0.02	0.508
Average response time (seconds)	1.22 ± 0.12	1.22 ± 0.12	0.927

^*∗*^Wilcoxon signed-rank test; logMAR = logarithm of the minimal angle of resolution.

## Data Availability

The clinical data used to support the findings of this study are restricted by the institutional review board of Keio University School of Medicine in order to protect patient privacy. Data are available from Prof. Kazuno Negishi (fwic7788@mb.infoweb.ne.jp) for researchers who meet the criteria for access to confidential data until November 30 , 2023.
